# The Role of Connectedness and Psychological Hardiness in EFL Teachers’ Pedagogical Effectiveness

**DOI:** 10.3389/fpsyg.2022.877057

**Published:** 2022-06-06

**Authors:** Zhenzhen Liu

**Affiliations:** School of Foreign Languages, Xinxiang Medical University, Xinxiang, China

**Keywords:** SLA, connectedness, psychological hardiness, EFL teacher, positive psychology, pedagogical effectiveness

## Abstract

The role of emotions in EFL teachers’ pedagogical success and efficacy has long been emphasized in the literature. However, the power and impact of teachers’ positive psycho-emotional factors like sense of connectedness and hardiness have been marginally explored in EFL contexts. Against this shortcoming, the present mini-review article made an attempt to provide the theoretical and research underpinnings of three prominent teacher-related factors, namely, connectedness, psychological hardiness, and pedagogical effectiveness as well as their interplay. Moreover, the research trends, gaps, and future directions are provided for enthusiastic researchers. Finally, some practical implications for EFL teachers, teacher trainers, and SLA researchers are offered to raise their awareness of psycho-emotional factors in second/foreign language education.

## Introduction

For a long time, teaching has been regarded as a relational job in which the teacher and students are both responsible for creating a successful instruction and learning process. Therefore, they need to work collaboratively to provide such optimal learning conditions (Xie and Derakhshan, 2021). In this profession, learning goes beyond simple exposure to a bunch of information; instead, it is a complicated process that includes social, psychological, and emotional interactions that establish a positive classroom connection (Strachan, 2020). This form of classroom connection between the teacher and students has been found to produce many positive academic outcomes including engagement, learning, motivation, success, achievement, wellbeing, and hope (Violanti et al., 2018; Wendt and Courduff, 2018; Frymier et al., 2019; Havik and Westergård, 2020; Derakhshan, 2021; Pishghadam et al., 2021; Xie and Derakhshan, 2021).

As pinpointed by Frisby (2019), this sense of relational closeness to students is an instance of positive interpersonal communication behaviors that adds vitality to the classroom milieu and fulfills different student needs. By definition, teacher–student connectedness refers to a feeling of belongingness and psychological affiliation in a classroom context that highlights the degree to which students feel personally accepted, valued, involved, and supported by others (Goodenow, 1993). In such a positive and caring classroom climate, teachers’ pedagogical performance and students’ academic engagement, wellbeing, achievement, interpersonal communication skills, sense of attachment, resilience, and the like are heightened (Quin, 2017; García-Moya, 2020). Another aspect that a positive teacher–student connection can improve in teachers is their psychological hardiness level in teaching as one of the most demanding jobs in the world. The construct of psychological hardiness has been the focus of substantial investigation in the past decades (Quick et al., 1997; Judkins et al., 2020). It is a personal trait referring to one’s capability to handle and respond to stressful events using proper coping strategies that convert unfavorable situations into learning opportunities (Maddi, 2004). Thus, it can be viewed as a mindset by which one views complications, difficulties, and stressful circumstances as personal challenges instead of road barriers of personal growth. Hardiness has been identified to influence teachers’ instructional performance under pressure, motivation, perfectionism, burnout level, resilience, optimism, and decisiveness (Cole et al., 2004; Erkutlu, 2012; Tokhmehforoushan Khiabani and Hadidi Tamjid, 2017).

Furthermore, psychological hardiness and a strong classroom connection between the teacher and his/her students can improve teachers’ affectivity as well (Tarajová and Metruk, 2020). As the most significant characteristic of high-quality education, teaching and teacher effectiveness refers to a composite of different good features in teachers that enrich students’ personal and academic life and associated aspirations (Day, 2012; Stronge, 2018). Effectiveness is far beyond simple pedagogical knowledge, but a meta-construct that includes a wide range of positive characteristics, competencies, and behaviors in teachers (Kwangsawad, 2017). Trying to clarify the characteristics of effective teachers, various scholars, such as Giovannelli (2003), Borg (2006), Xuerong (2012), and Ko (2014), used different research tools to provide lists of features representative of effective teachers. A summary of such characteristics includes teachers’ useful classroom strategies, interactions, student involvement, emotion sensitivity, clear lesson presentation, professional and friendly classroom management and atmosphere, proper feedback provision, having pedagogical clarity, and being expert in curriculum content and the required teaching techniques. Despite these attempts, this line of research has mostly revolved around the exploration of effective teacher characteristics from students and teachers’ perspectives and its association to positive psych-emotional constructs like connectedness and psychological hardiness has been overlooked, to date. To fill this gap, this mini-review study tried to present the theoretical and practical aspects of two critical interpersonal communication skills (connectedness, psychological hardiness) and EFL teachers’ effectiveness making references to the existing gaps and future directions in this domain.

## Background

### The Origins and Definitions of Connectedness

The concept of connectedness or a sense of belongingness and kinship to a specific individual or group was first introduced in the context of school in the late 1990s. Its history has never been solid and straightforward as “connectedness” was mixed up with other cognate terms (Baumeister and Leary, 1995; García-Moya, 2020). Connectedness has mainly been defined as a sense of affinity and belongingness in school contexts, while some scholars considered it as a component of other related terms like “relatedness” (Hagerty et al., 1993). Later, the concept became more consolidated when it was associated with caring about individual learners and their learning (Blum and Libbey, 2004). This led to the expansion of literature in this area that culminated in the introduction of other related concepts, such as belonging, bonding, and engagement. During this period, little progress was made as various definitions and operationalizations were offered for the construct of connectedness in education (Wang and Degol, 2016). Now the concept of connectedness is understood and situated in the school climate framework that comprises four dimensions of the academic environment, institutional environment, relationships, and safety (Wang and Degol, 2016). Based on this framework, connectedness is a crucial element of the third dimension; community relationship.

Despite these advances, research in this domain is still laden with interchangeable uses of connectedness, bonding, belonging, and engagement that is in contrast with the current literature. To obtain higher conceptual clarity in this line of inquiry, it is believed that the mentioned terms must be reflected upon, especially their commonalities and differences, to facilitate more scientific progress in this area and provide a coherent evidence base (García-Moya, 2020).

### Characteristics of Connected Teachers

In the educational arena, teachers who strive to establish a sense of “connectedness” with their pupils have a number of common features (García-Moya, 2020). The first characteristic of such teachers concerns their attempts to develop a friendly, positive, and democratic classroom climate (Chhuon and Wallace, 2014). They also seek opportunities for out-of-class interactions in the form of informal greetings, sports, and sharing a lunchtime with students (Neely et al., 2016). Connected teachers frequently use jokes and open communication strategies that reduce the physical and emotional distance in the class (Yu et al., 2018). Another feature of connected teachers is their personalized, respectful, and humanized type of academic relationship with students represented with high empathy and willingness to listen to problems (García-Moya, 2020). Depending on the context and academic objectives, this list of features can be expanded by running further studies including the voices of different stakeholders.

### Contributions of Connectedness to L2 Education

A classroom culture/climate that is oriented to a sense of proximity and closeness between the teacher and students can bring about many optimal outcomes for teachers and students (Wendt and Courduff, 2018). It can improve teachers’ pedagogical performance, classroom management skills, wellbeing, positive interpersonal communication abilities, and teaching motivation (Quin, 2017; García-Moya, 2020; Xie and Derakhshan, 2021). Moreover, it can contribute to students’ academic engagement, wellbeing, achievement, interpersonal communication skills, sense of attachment, resilience, learning, motivation, success, and hope (Violanti et al., 2018; Frymier et al., 2019; Havik and Westergård, 2020; Pishghadam et al., 2021). Due to the complex and emotional nature of second/foreign language education, the construct of connectedness can generate many other positive consequences like psychological hardiness and effectiveness among EFL teachers, too. However, limited research has been done on such contributions drawing on the ideas of positive psychology.

### Psychological Hardiness: Conceptualization and Related Terms

The concept of psychological hardiness is conceptualized as an individual capacity to positively respond to negative events and stressors in life and career (Maddi, 2004). As put by Hiver and Dörnyei (2017), hardiness is an ability to fight and minimize the negative effects of stress on one’s performance. It includes three senses or attitudes of *commitment*, *control*, and *challenge* (Sheard and Golby, 2010). Commitment refers to the predisposition to deeply engage oneself in what one is doing or facing. Control concerns one’s propensity to perceive and act as if he/she plays a role in different life incidents rather than being a helpless person. Finally, challenge refers to the belief that change is quite common in one’s life and the expectation of such variations is useful for personal growth. These attitudes are in tune with the challenging nature of L2 education in which EFL teachers and students have to deal with linguistic, intercultural, and psycho-emotional factors at the same time. Other than having a sufficient pedagogical knowledge base, EFL teachers need to be psychologically hard in the complex and challenging system of L2 education. This shift toward teacher psychology and emotions is rooted in PP that focuses on the power of positive emotions in education and development (MacIntyre et al., 2019).

Consistent with these progressions, some similar terms have been proposed for psychological hardiness including resilience, buoyancy, coping, and immunity. Although they seem equivalent to hardiness, they differ on some grounds. For instance, resilience refers to one’s ability to locate and tackle general life challenges and complications, while buoyancy is more specific and concerns academic adversities that happen in one’s academic life (Martin and Marsh, 2019). Moreover, coping pertains to specific techniques and strategies that one uses to solve problems. As the last cognate, immunity refers to defensive mechanisms that a person utilizes to decrease and inhibit the challenges and harms to his/her motivation, identity, behavior, and practice (Hiver, 2017). It should be noted that, in many cases, the boundaries of these similar terms are not clear-cut; hence, their interchangeable use continues even in the current era.

### Teacher and Teaching Effectiveness: Models and Trends

The first stones of teacher/teaching effectiveness were laid in 1970s by *the process-product model* that proposed some important personality-related and teaching-related factors contributing to effective teaching (Cooper and McIntyre, 1996). Then, *the professional craft knowledge approach* came into vogue considering teaching as the ability to analyze particular situations and implement that craft knowledge in different contexts (Cooper and McIntyre, 1996; Soodmand Afshar and Doosti, 2014). With advances in L2 language education, especially *post-method* and *communicative approaches*, the trends in viewing effectiveness shifted toward “specificity” and “particularity” proposed by *the constructivist approaches*. In this era, each teacher was seen as unique with particular systems of teaching beliefs and values that produced reflective teachers. In this approach, reflection was one of the most essential features of effective teachers (Richards and Farrell, 2005). Trying to complement these perspectives, more explorations in various contexts were made that highlighted the prominent role of content knowledge, pedagogical knowledge, socio-cultural factors, and personality-related factors influencing effectiveness (Soodmand Afshar and Doosti, 2014).

Furthermore, teaching profession was regarded as both a science and art in that it needs research findings on pedagogy and learning as well as creativity and talent on the part of the teacher (Wang, 2017). This mysterious nature of teaching and its direct impact on learning led to a surge of international interest in unpacking the characteristics of effective teaching/teachers in the context of L2 education listing various features. The commonality of these studies and their identified characteristics is that effective teachers have a good command of the target language, are good communicators, have interpersonal skills, can present the lesson meaningfully and excitingly, use group work to increase learner involvement, can establish a friendly and supportive learning contexts, consider students’ emotions, and tolerate classroom errors (Borg, 2006; Lee, 2010; Chen, 2012; Kourieos and Evripidou, 2013; Ko, 2014; Stronge, 2018). Although these attempts are insightful for EFL/ESL teaching and learning, there are many other avenues in this area that are left unexplored. The following part presents research findings and evidence in teacher effectiveness.

### Researching L2 Teacher Effectiveness

With the wide acceptance of the claim that teaching effectiveness leads to learning effectiveness, the construct of teacher effectiveness found its way in SLA research and practice (Borg, 2006; Day, 2012; Stronge, 2018). After its establishment, the concept was given a prime significance in EFL/ESL contexts with numerous studies focusing on what the term means and what its indicators might be from students’ and teachers’ standpoints. The leading researchers in this domain, have mostly used questionnaires, semi-structured interviews, expert interviews, and observations in order to disclose the characteristics of EFL teachers (Borg, 2006; Chen, 2012; Xuerong, 2012; Ko, 2014, among others). It is surprising that SLA researchers are still obsessed with the indicators of this construct using a limited number of research instruments. To reach a more vivid picture, L2 scholars could have used diaries and portfolios to capture the dynamism of teacher effectiveness. They could also include the voices of other stakeholders in defining “effectiveness” and the “features of an effective EFL teacher.” The role of positive emotions and democratic classroom climate that create a sense of intimacy and connectedness in teachers in the face of inherent challenges of L2 education have been largely ignored, thus far. Moreover, correlations of teacher effectiveness and many psycho-emotional variables in L2 education, such as intelligence, language competence, resilience, motivation, burnout, perfectionism, immediacy, clarity, and the like, are recommended to future researchers.

## Concluding Remarks

In this mini-review article, it was argued that EFL teachers’ sense of connectedness with their pupils and their psychological hardiness in managing and encountering academic setbacks of L2 teaching is highly influential in raising their pedagogical effectiveness. In other words, a positive classroom culture/climate where there is a strong rapport between the teacher and his/her students helps EFL teachers when facing L2 education challenges. This, in turn, contributes to resilience and buoyancy among the teachers that bring about many positive outcomes including teaching and learning effectiveness. Hence, the study can be beneficial to EFL teachers in that it increases their awareness of the power of positive emotions in L2 education. They can use the ideas in this study and establish a classroom environment in which both students and teachers feel connected and psychologically tough in the face of setbacks that ultimately generate academic success. Likewise, EFL teacher educators can use this review and offer professional development courses to teachers raising their knowledge and practice of positive emotions in the classroom so that their teaching effectiveness is improved. They can educate novice teachers that L2 instruction is not all about teachers’ pedagogical knowledge but an ocean of psycho-emotional factors. Finally, SLA researchers can find this study helpful and make efforts to bridge the existing gaps in this area. Most of the related studies on teachers’ connectedness, hardiness, and effectiveness have used one-shot designs *via* questionnaires and single-session interviews that are inconsistent with the dynamic nature of these teacher-psychology variables. Therefore, more qualitative and longitudinal studies are recommended to enthusiastic researchers. The element of culture is limitedly (if any) considered in this line of inquiry, so cross-cultural studies can add informative ideas to the body of knowledge in this domain. The correlation between teachers’ demographic information (age, gender, experience, academic degree, major, etc.) and the three variables covered in this article is also suggested. Lastly, this study can be complemented by similar studies using other variables proposed in PP, such as interpersonal communication skills, love, hope, joy, resilience, passion, commitment, closeness, engagement, open-mindedness, willingness to communicate, ambiguity tolerance, and so on.

## Author Contributions

The author confirms being the sole contributor of this work and has approved it for publication.

## Conflict of Interest

The author declares that the research was conducted in the absence of any commercial or financial relationships that could be construed as a potential conflict of interest.

## Publisher’s Note

All claims expressed in this article are solely those of the authors and do not necessarily represent those of their affiliated organizations, or those of the publisher, the editors and the reviewers. Any product that may be evaluated in this article, or claim that may be made by its manufacturer, is not guaranteed or endorsed by the publisher.
